# Preservation of the round ligament to accommodate transient portal hypertension after major hepatectomy

**DOI:** 10.1007/s00423-022-02581-x

**Published:** 2022-06-08

**Authors:** D. Koliogiannis, H. Nieß, V. Koliogiannis, M. Ilmer, M. Angele, J. Werner, M. Guba

**Affiliations:** 1 Department of General, Visceral and Transplant Surgery, LMU Klinikum, Campus Grosshadern, Marchioninistrasse 15, 81377 Munich, Germany; 2 Department of Radiology, LMU Klinikum, Munich, Germany

**Keywords:** Round ligament preservation, Major liver resection, Portal hypertension, Posthepatectomy liver failure

## Abstract

**Purpose:**

Posthepatectomy liver failure (PHLF) remains a leading cause of death after extensive liver resection. Apart from the size and function of the remaining liver remnant, the development of postresection portal hypertension (pHT) plays a crucial role in the development of PHLF. We hypothesize that the umbilical vein in the preserved round ligament (RL) may recanalize in response to new-onset pHT after extended hepatectomy, thus providing a natural portosystemic shunt.

**Methods:**

In this exploratory study, RL was preserved in 10 consecutive patients undergoing major liver resection. Postoperative imaging was pursued to obtain evidence of reopened umbilical vein in the RL. The postoperative course, including the occurrence of PHLF, as well as the rate of procedure-specific complications were recorded.

**Results:**

None of the 10 cases presented with an adverse event due to preservation of the RL. In 6 cases, postoperative imaging demonstrated reopening of the umbilical vein with hepatofugal flow in the RL. The rates of procedure-related surgical complications were lower than would be expected in this population; in particular, the rate of occurrence of PHLF as defined by the International Study Group of Liver Surgery (ISGLS) was low.

**Conclusion:**

Our results support the theoretical concept of portosystemic pressure relief via a preserved umbilical vein after major liver surgery. As preservation of the RL is easily done, we suggest keeping it intact in extended hepatectomy cases and in patients with preexistent pHT.

## Introduction

Posthepatectomy liver failure (PHLF) remains a feared complication after extensive hepatectomy. The main cause of PHLF is too small residual liver or inadequate liver function. Loss of liver parenchyma drastically reduces the cross-sectional area of the portal vein, which can lead to acute portal hypertension (pHT) and in turn may exacerbate PHLF. This hyperperfusion syndrome has long been known as “small for size” in partial liver transplantation, and severe vascular shear stress associated with endothelial dysfunction has been identified as one of the major underlying pathomechanisms [1, 2]. Furthermore, this hypothesis is supported by the fact that small-for-size syndrome can be successfully treated by interventions aimed at reducing portal hypertension, such as porto-caval shunting [3], splenic artery ligation [4], or splenectomy [5]. The role of portal hypertension after liver resection has been studied primarily in the context of resections for cirrhosis but is gaining attention as a risk factor for PHLF in extended resections for noncirrhotic livers [6].

The round ligament (Lig. teres hepatis) is a fibrous cord resulting from the obliteration of the umbilical vein. In cirrhotic patients, the umbilical vein may be recanalized forming porto-systemic shunts around the umbilicus, known as Caput medusa [7].

We hypothesize that recanalization of the veins in the round ligament (RL) may also occur in situations of acute portal hypertension to alleviate portal pressure. In a case series of 10 consecutive major liver resections, we investigated the feasibility and the early outcomes when the RL is preserved.

## Methods

This exploratory study included 10 consecutive patients from our institution who underwent major liver resection between June 2021 and October 2021. Basic patient characteristics, procedure-specific details, and early postoperative course until hospital discharge were recorded. The occurrence and severity of PHLF were classified according to the definition for PHLF by the International Study Group of Liver Surgery (ISGLS) [8], surgical complications according to the Clavien-Dindo classification [9]. Routine imaging required prior to the surgical procedure was available for evaluation. Postoperative imaging was limited to cross-sectional imaging procedures that were otherwise indicated; in some cases, examinations could be supplemented by noninvasive Doppler sonography.

Anonymized analysis of data from patients undergoing liver surgery was covered by broad consent approved by the institution’s local ethics committee (no. 19–395). All investigations complied with the principles of the Declaration of Helsinki (64th WMA General Assembly, Fortaleza, Brazil, October 2013).

### Patients

Patients were men and women aged between 35 and 78 years, suffering from intrahepatic malignancies or hepatic metastases due to colorectal carcinoma. One patient was diagnosed with hemangioma. Criterion of exclusion from the study was the need for resection of the RL, e.g., in case of left hepatectomy.

### Statistical analysis

All statistical analysis were performed using the Statistical Package for Social Sciences (Version 27.0; SPSS Inc, Chicago, IL, USA) and GraphPad Prism (Version 9.0.0; GraphPad Software, San Diego, CA, USA).

### Operative procedure

**Fig. 1:**
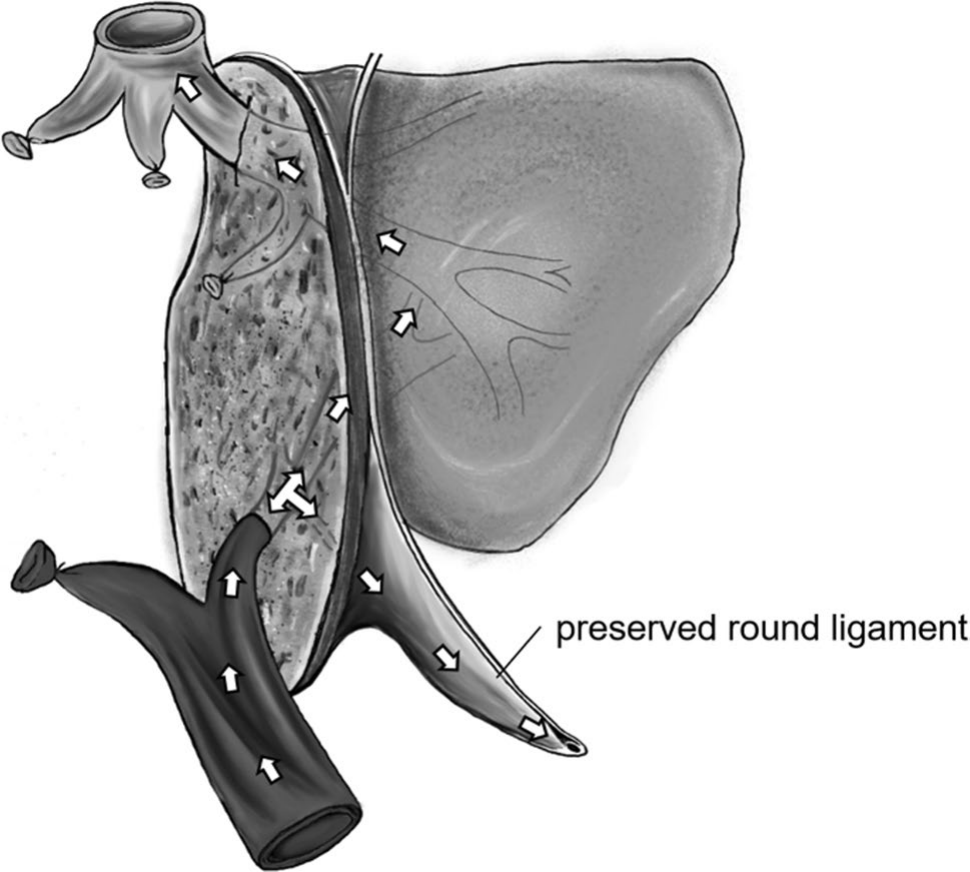
Operative situs showing the preservation of the round ligament. Arrows indicate the potential shunt flow

**Fig. 2:**
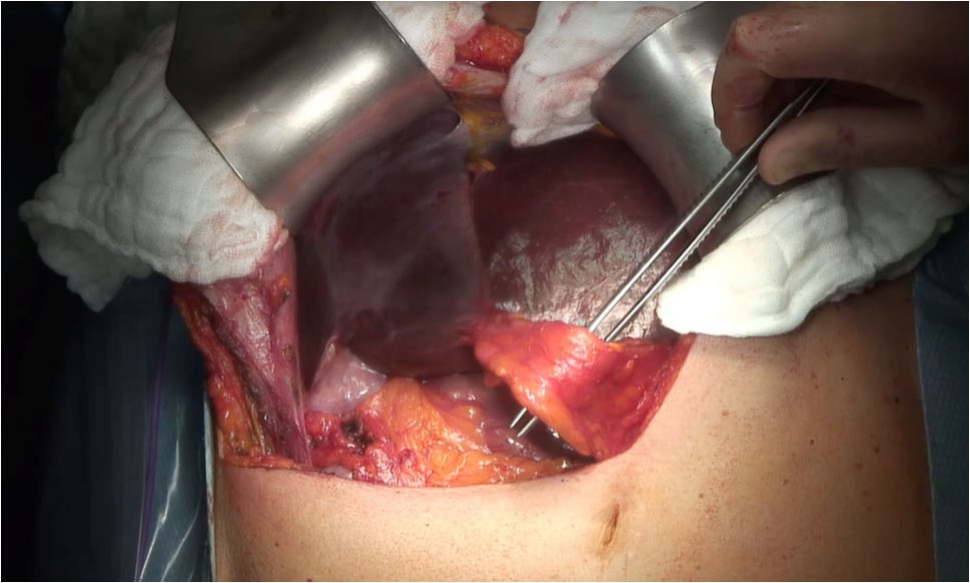
Intraoperative situs showing the preservation of the round ligament

During standard liver resections, the round ligament is traditionally cut and ligated. In this study, we have dissected the RL from the peritoneum on the right side and entirely preserved the ligament down to the umbilicus (see Figs. 1 and 2). Whenever necessary, the falciform ligament was divided, taking care not to affect the structures of and around the RL.

Parenchymal dissection was performed with either LigaSure™ tissue fusion, ultra-sonic (Söring™) tissue dissection or vascular staplers (Covidien™). In all patients, 2 Robinson drains were placed at the resection site of the liver and below the hepatic ligament. On the second postoperative day, the drains were removed if the drain contents were unremarkable.

## Results

### Patients

Major liver resection was scheduled in 4 patients due to hepatocellular carcinoma, in 3 cases due to cholangiocellular carcinoma or gallbladder cancer, in 2 patients due to colorectal liver metastases, and in one patient due to hemangioma. In 8 patients, right or extended right hepatectomy was performed, one patient underwent resection of liver segments 5 and 6, and one patient underwent central trisegmentectomy of the liver in addition to atypical resection in liver segments 3 and 7. Five of the patients reported showed normal liver parenchyma, and the others were suffering from steatosis, fibrosis, and in one case cirrhosis. One patient with a HCC showed preoperatively mild clinical signs of pHT. For more details on patients’ characteristics, see Table 1 and Table 2.

**Table 1:** Patients’ characteristics

	(Mean with SEM)	
Gender (*n*)	♂: 6	♀: 4
Age (years)	58.9 ± 4.21	
BMI (kg/m^2^)	23.7 ± 1.09	
ASA score	3.0 ± 0.0	
LabMELD	6.5 ± 0.50	
Child–Pugh score	5.6 ± 0.31	
ICU stay (days)	2.4 ± 0.60	
Length of stay (days)	15.1 ± 1.59

**Table 2:** Preoperative characteristics and postoperative findings of the study population (cases with reopened RL are indicated in bold type)

Patient	Indication	Liver parenchyma	Preoperative PHT*	Procedure	Remnant liver volume in cm^3^ (in %)	Complication (Clavien-Dindo)	Renal failure	PHLF**	Reopened RL
1	HCC	Cirrhotic	Yes	Right hepatectomy	774 (39.3)	None (1)	No	No	**Yes**
2	HCC	Fibrotic	No	Right hepatectomy	305 (19.7)	Incisional hernia (3b)	Yes; no dialysis	**Grade A**	**Yes**
3	Gallbladder cancer	Normal	No	Right hepatectomy	305 (20.6)	None (1)	No	No	**Yes**
4	Hilar cholangiocarcinoma	Normal	No	Extended right hepatectomy	666 (38.7)	None (1)	No	No	**Yes**
5	HCC	Fibrotic	No	Extended right hepatectomy + atyp. seg. 2/3/4b	613 (22.1)	None (1)	No	No	**Yes**
6	Gallbladder cancer	Fibrotic	No	Seg. 5/6 resection	1622 (77.2)	None (1)	No	No	Not proven
7	CRC-M	Steatotic	No	Central trisegmentectomy + atyp. 3 and 7	678 (30.3)	None (1)	No	No	Not proven
8	CRC-M	Normal	No	Extended right hepatectomy	636 (36.7)	None (1)	No	No	Not proven
9	Hemangioma	Normal	No	Right hepatectomy	756 (21.3)	Pleural effusion (3a)	No	No	Not proven
10	HCC	Normal	No	Right hepatectomy	722 (30.7)	Pleural effusion (3a)	No	No	**Yes**

^*^One of the following: ascites, splenomegaly, gastroesophageal varices, thrombocytopenia < 100,000/ml; **according to the definition by the International Study Group of Liver Surgery (ISGLS)[8]

### Recanalization of the umbilical vein

**Fig. 3:**
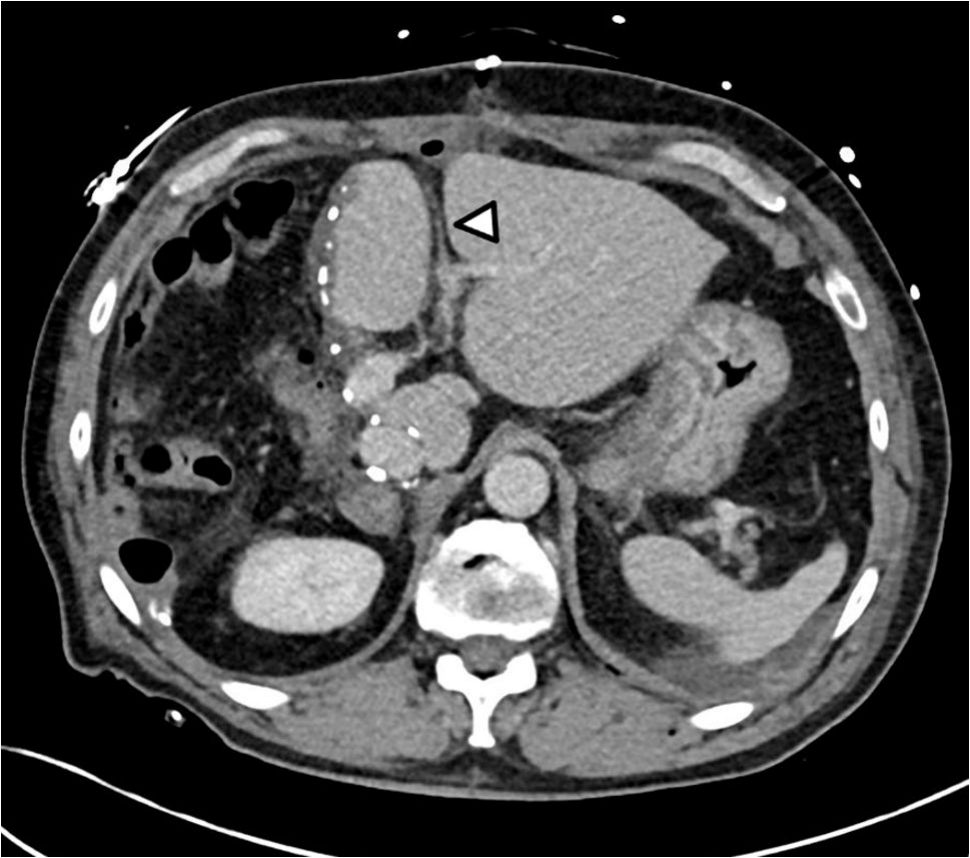
CT scan showing the postoperative recanalization of the umbilical vein. Arrow indicates the reopened umbilical vein

**Fig. 4:**
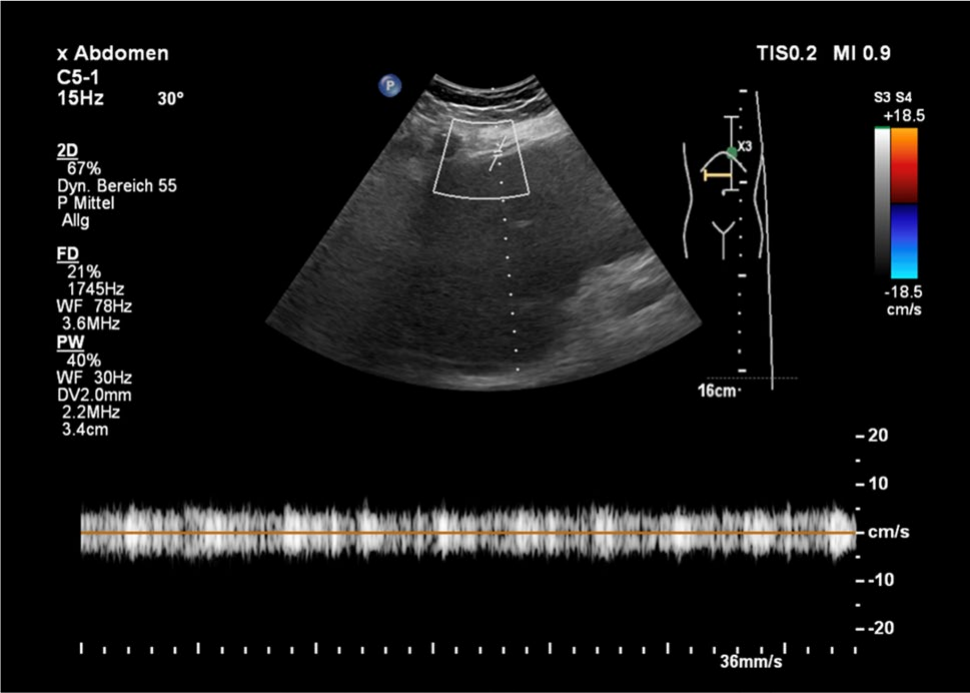
Postoperative Doppler ultrasound showing hepatofugal flow in the round ligament

In 6 patients, we could confirm recanalization of the umbilical vein by Doppler ultrasound or contrast-enhanced cross-sectional imaging, exemplified in Figs. 3 and 4.

### Outcome

In the 10 consecutive patients, we observed a perioperative mortality rate of 0%. We observed one surgical complication (Clavien-Dindo grade 3b). Two patients developed right-sided pleural effusion requiring drainage (Clavien-Dindo grade 3a). One out of 10 patients showed mild postoperative liver failure (grade A) according to the definition for PHLF by the International Study Group of Liver Surgery (ISGLS) [8]. The median postoperative length of stay in the study cohort was 12 days.

## Discussion

Posthepatectomy liver failure remains the leading cause of early mortality after extended liver resection. Portal hypertension plays a critical role in the development of PHLF. Although pHT is required to some extent to stimulate liver regeneration, excessive pHT may have adverse effects on liver function [10]. Portal hyperperfusion as a consequence of pHT may lead to congestion and endothelial damage due to excessive vascular shear stress. Portal hyperperfusion also negatively affects arterial flow in the liver remnant, leading to parenchymal necrosis and ischemic cholangitis [11].

The role of new-onset portal hypertension has been extensively studied in partial liver transplantation but came late into the fore as a potential pathomechanism for PHLF. In major liver resection in noncirrhotic patients, Allard et al. have shown that post hepatectomy portal vein pressure predicts LF and mortality [6]. Bogner et al. have shown that, particularly, the increase of portal venous pressure after major hepatectomy may be predictive for PHLF [12].

Based on the experience with patients with advanced liver cirrhosis and long-lasting pHT, in whom the umbilical vein is frequently recanalized, we hypothesize that this mechanism may also occur in cases of acute pHT after extended liver surgery. Another hint that preservation of the RL may alleviate pHT comes from liver surgery in cirrhotic patients [13]. Patients undergoing laparoscopic liver resection, in which the RL is usually kept intact, develop less postoperative ascites than patients undergoing open liver surgery, in which the RL is usually divided.

Traditionally, the RL is deliberately dissected to facilitate mobilization of the liver. However, to allow collateralization via the umbilical vein, the RL must remain intact. In our experience, mobilization of the liver can be easily done even with the RL intact, so dissection is not necessary.

To our knowledge, this is the first case series describing RL preservation during major hepatectomy. The results of our 10 first cases studied here did not reveal any adverse events due to preservation of the RL; on the contrary, the complication rate was lower than what could be expected given the extent of the surgery. In addition, we were able to detect recanalization of the RL early after surgery in 6 of 10 patients in our case series, thus providing circumstantial evidence that the porto-systemic shunt via the umbilical vein may serve as a physiologic pressure relieve for sudden onset pHT. Recruitment of nonphysiologic portosystemic shunts, e.g., porto-renal, in response to short-acting PHT was not apparent in the available postoperative imaging. At this point, it is, of course, speculative to attribute the good outcome to preservation of the RL. However, the lack of negative implications for liver resection and the potential to accommodate a new-onset pHT, thereby improving patient outcome, justifies the preservation of the RL in any case.

The main limitation of this case series is the small number of cases and the lack of an appropriate control group. Another important limitation is that we failed to systematically measure portal venous pressure before and at regular intervals after liver surgery. This shortcomings certainly need to be systematically addressed in a randomized controlled trial on this topic using the least invasive procedures possible, such as Doppler sonography and/or MR angiography.

## Conclusion

Our study provides circumstantial evidence for a theoretically plausible hypothesis that preservation of the RL may help alleviate posthepatectomy pHT and thus prevent PHLF. Based on our excellent experience and the fact that preservation of the RL does not interfere with the procedure, we suggest that the RL should be preserved in extended liver surgery and especially in patients with preexisting pHT.
